# Detectable Levels of Bacterial Pathogens in the Rivers of the Lake Chaohu Basin, China

**DOI:** 10.3390/ijerph16234857

**Published:** 2019-12-03

**Authors:** Keqiang Shao, Xin Yao, Guijuan Xie, Yuanyuan Wu, Yang Hu, Xiangming Tang, Guang Gao

**Affiliations:** 1 Taihu Laboratory for Lake Ecosystem Research, State Key Laboratory of Lake Science and Environment, Nanjing Institute of Geography and Limnology, Chinese Academy of Sciences, Nanjing 210008, China; kqshao@niglas.ac.cn (K.S.); xeiguijuan@126.com (G.X.); chaohus@126.com (Y.H.); xmtang@niglas.ac.cn (X.T.); 2 School of Environment and Planning, Liaocheng University, Liaocheng 252000, China; yaoxin@lcu.e.du.cn; 3 University of Chinese Academy of Sciences, Beijing 100049, China; 4 Sino-Japan Friendship Center for Environmental Protection, Beijing 100029, China; whw7018@163.com

**Keywords:** Illumina miseq sequencing, Lake Chaohu, opportunistic pathogens, river

## Abstract

Bacterial pathogens are one of the causes of human diseases and have a serious impact on environmental health. In this study, we investigated the bacterial pathogen community in 88 sites at rivers around Lake Chaohu Basin, China, using Illumina miseq sequencing. The results showed that three opportunistic pathogens: *Acinetobacter*, *Massilia*, and *Brevundimonas*, were the three abundant bacterial genera in all samples, and had a relative abundance of 0.33 to 49.28% (average 8.80%), 0.06 to 25.4% (average 4.6%), 0.01 to 12.82% (average 2.6%) of all bacterial sequences, respectively. Our results indicated that a high abundance of opportunistic pathogens was observed in the rivers of the Lake Chaohu Basin, and that effective treatment and monitoring of sewage entering into rivers should be further strengthened.

## 1. Introduction

Rivers are the primary receivers of organic matter and nutrients from terrestrial ecosystems [1], and provide key coupling of biogeochemical cycles in aquatic ecosystems [2]. Furthermore, rivers are important sources of renewable water for humans and freshwater ecosystems [3,4]. However, the release of effluent from wastewater treatment plants, ineffective septic tank systems, and storm water runoff may cause the direct inflow of sewage and excrement bacteria into rivers [5]. These bacterial genera usually include waterborne pathogens that are a danger to human health [6,7,8]. Therefore, understanding the bacterial pathogen community in the river is of great importance. 

Lake Chaohu (31°25′–31°43′ N, 117°16′–117°51′ E) is the fifth largest freshwater lake in China and is located at the center of Anhui Province, downstream of the Yangtze River. The lake has a surface area of 760 km^2^ and a mean depth of 2.69 m. The lake can be divided into two regions: from the Zhongmiao Temple to Qitouzui Cape, the eastern region is mesotrophic, and the western region is eutrophic to hypertrophic. The eastern lake region connects to the Yuxi River, which is the only channel connecting the eastern lake to the Yangtze River, permitting water exchange. The western lake region receives major inflows including the Nanfei and Shiwuli Rivers (both have sewage outfalls), the Hangbu, and the Pai River (which contributes the greatest discharge) [9,10]. These western rivers account for almost 60% of the total runoff volume contributed annually to the lake [9,10]. The aim of our study was to examine the bacterial pathogen community in the rivers around the Lake Chaohu Basin using Illumina Miseq sequencing. 

## 2. Materials and Methods

**Figure 1:**
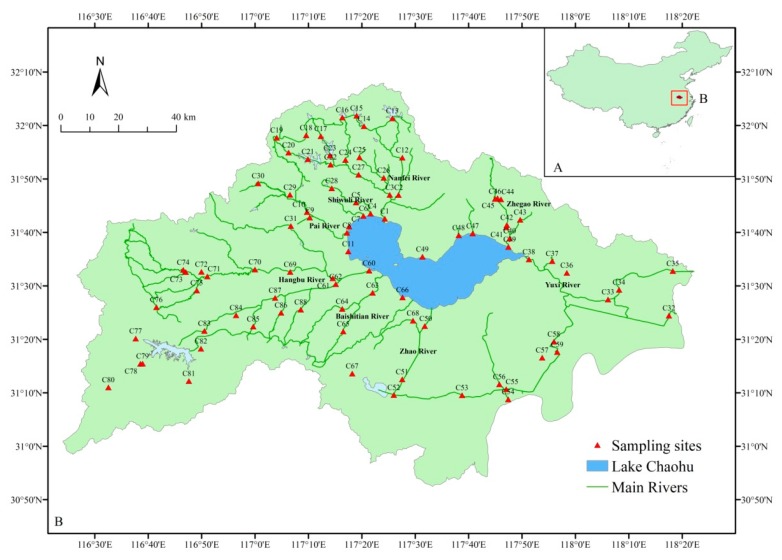
Map showing the location of the 88 sampling sites in rivers of the Lake Chaohu Basin.

On 15 February 2018, we carried out field work at 88 sites of the rivers around this lake (Figure 1). At each site, surface water (top 50 cm) was collected with a 5 L Schindler sampler. For 16S rRNA gene analysis, a subsample of water (500 mL) was pre-filtered in situ with a 0.2 μm pore-size polycarbonate membrane (47 mm diameter, Millipore) using a hand-driven vacuum pump. These filters were frozen at −80 °C until DNA extraction was performed. The remaining water samples were transported to the laboratory in dark cooling boxes, and processed 3–5 h after sampling within 4 h for immediate physicochemical analysis.

The total DNA was extracted using proteinase K, sodium dodecy1 sulfate, and cetyltrimethyl ammonium bromide, follow by phenol-chloroform extraction and isopropanol precipitation [11]. Crude DNA extracts were then purified by the E.Z.N.A^®^ cycle-Pure kit (Omega Bio-Tek Inc., Norcross, GA, USA). The V4–V5 regions of the 16S rRNA genes were amplified using the primers 515F (GTGCCAGCMGCCGCGGTAA) and 907R (CCGTCAATTCMTTTRAGTTT). The sequencing service was performed by an Illumina Miseq platform at Personal Biotechnology Co. Ltd. (Shanghai, China). Sequence reads (subsequently referred to as ‘reads’) were processed using the Quantitative Insights Into Microbial Ecology (QIIME) v. 1.8.0 pipeline [12]. After demultiplexing, quality filtering, denoising, and chimera removal, bacterial phylotypes were identified and assigned to operational taxonomic units (OTUs, 97% cutoff) using the Uclust algorithm [13] to generate final OTUs. The longest sequence in each cluster was chosen to be the representative sequence, and sequences were annotated by the Silva rRNA database project [14]. The raw pyrosequencing data we generated were submitted to the National Center for Biotechnology Information (NCBI) Sequence Read Archive, under accession number SRP189003. 

## 3. Results and Discussion

**Figure 2:**
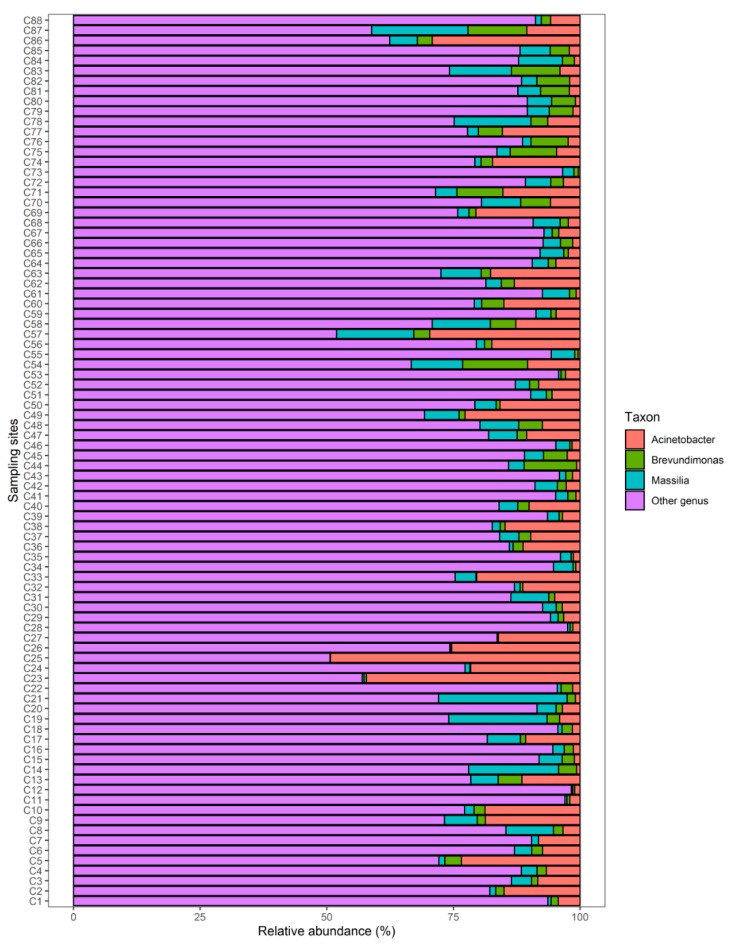
Relative abundances of *Acinetobacter*, *Massilia*, and *Brevundimonas* in the 88 sampling sites of the rivers in the Lake Chaohu Basin.

All water samples were slightly alkaline, with pH values ranging from 7.60 to 9.66 (mean = 8.56), and the content of total nitrogen and total phosphorus ranged from 0.71 to 18.80 mg L^−1^ (mean = 4.00 mg L^−1^) and 0.03 to 3.00 mg L^−1^ (mean = 0.22 mg L^−1^), respectively (Table 1). Three bacterial pathogens: *Acinetobacter*, *Massilia* and *Brevundimonas*, were the three abundant bacterial genera in all samples, and had a relative abundance of 0.33 to 49.28% (average 8.80%), 0.06 to 25.4% (average 4.6%), 0.01 to 12.82% (average 2.6%) of all bacterial sequences among the 88 samples, respectively (Figure 2). Examination of the scientific literature showed they are opportunistic pathogens and infectious [15,16,17]. An opportunistic pathogen is defined as one that usually causes disease only when the host immune system is weakened [15]. As newer pathogens, *Acinetobacter* plays an important role in the colonization and infection of patients admitted to hospitals [16]. Some species of *Massilia* are known to cause infections in immunocompromised patients [18], but methods of identification are still insufficient [17]. *Brevundimonas* may be a more widespread pathogen than was hitherto thought, causing infections by being invasive [19]. The high abundance of *Acinetobacter*, *Massilia*, and *Brevundimonas* in this study may be associated with failure in sewage treatment processes, which may be reflected in the high TN and TP content of the river water environment. Previous studies have also shown that the occurrence of pathogenic bacteria in river water may increase near large urban populations following failure in sewage treatment processes [6,7]. Opportunistic pathogens are typically characterized as organisms that can become pathogenic following a perturbation to their host [20]. Furthermore, humans infected with opportunistic pathogens harboring antibiotic resistance genes result in increased difficulty of treatment [15,20]. Therefore, a mass of opportunistic pathogens from rivers could spread to the whole of Lake Chaohu, causing serious environmental health risks. Furthermore, effective treatment and monitoring of untreated domestic wastewater around the Lake Chaohu Basin is of paramount importance and should be further strengthened.

## 4. Conclusions

In conclusion, a high abundance of the opportunistic pathogens *Acinetobacter*, *Massilia*, and *Brevundimonas* was observed in the rivers of the Lake Chaohu Basin, and effective treatment and monitoring of sewage entering into rivers should be further strengthened.

## Figures and Tables

**Table 1:** Mean, maximum, and minimum values for the three physicochemical parameters for the 88 sampling sites in the rivers around the Lake Chaohu Basin. Abbreviations are TN (total nitrogen), TP (total phosphorus).

Physicochemical Parameters	Mean	Range
pH	8.56	7.60–9.66
TN (mg L^−1^)	4.00	1.24–18.80
TP (mg L^−1^)	0.22	0.03–1.47
